# Editors’ Book Table

**Published:** 1872-04

**Authors:** 


### Editors' Book Table.

[NOTE. — All works reviewed in the columns of the CHICAGO MEDICAL JOURNAL may be found in the extensive stock of W. B. KEEN, COOKE & CO., whose catalogue of Medical Books will be sent to any address upon request.]

*Injuries of Nerves and their Consequences.* By S. WEIR MITCHELL, M.D., Member of the National Academy of Sciences; Fellow of the Philadelphia College of Physicians, etc., etc. Philad.: J. B. Lippincott & Co. 1872.

*Lectures on Aural Catarrh; or the Commonest Forms of Deafness and their Cure.* (Mostly delivered at St. Mary's Hospital.) By PETER ALLEN, M.D., Fellow of the Royal College of Surgeons, Edinburgh; Member of the Royal College of Surgeons, England; etc., etc. New York: Wm. Wood & Co. 1872.

*Memoranda on Poisons.* By the late THOMAS HAWKES TANNER, M.D., F.L.S. Third and completely revised edition. Philad.: Lindsay & Blakiston. 1872.

*Dr. Rigby's Obstetric Memoranda.* Fourth edition, revised and enlarged. By ALFRED MEADOWS, M.D., Physician to the General Lying-in Hospital, and to the Hospital for Women, etc. Philadelphia: Lindsay & Blakiston. 1872.

*The Treatment of Venereal Diseases.* A Monograph on the Method pursued in the Vienna Hospital, under the direction of Prof. Von Sigmund; including all the formulæ. By M. H. HENRY, M.D., Surgeon to the New York Dispensary Department of Venereal and Skin Diseases; Fellow of the New York Academy of Medicine, etc., etc., etc. Adapted and arranged from the German. New York: Wm. Wood & Co. 1872.

### PAMPHLETS.

*Annual Address.* Delivered before the Cleveland Homeopathic Hospital College, February 14th, 1872. By J. D. BUCK, M.D., Professor of Psychology.

*Seventh Annual Report of the Institution for the Education of Feeble-Minded Children.* Located at Jacksonville, Illinois. December, 1871.

*Remarks on Strictures of the Urethra of Extreme Calibre, with Cases, and a Description of New Instruments for their Treatment.* By F. N. OTIS, M.D., Clinical Professor of Venereal Diseases in the College of Physicians and Surgeons, New York, etc. Reprinted from the New York Medical Journal, February, 1872.

*On the Physiology of Syphilitic Infection.* By FESSENDEN N. OTIS, M.D., Clinical Professor of Venereal Diseases at the College of Physicians and Surgeons, New York; etc., etc. Reprinted from the Medical Gazette.

*Report of the Meeting for the Organization of a Territorial Medical Society*, held in Denver, Colorado, Sept. 19, 1871, including Constitution and By-laws adopted by the meeting.

*The Mysterious Death of Margaret Campbell critically examined; with a Review of the Testimony, Verdict of the Jury, Comments of the Press, etc. Exitus acta probat*, (Ovid.) By T. D. CROTHERS, M.D., Member of the Albany County Medical Society, etc.

*Proceedings of the Twenty-first Annual Meeting of the New York State Homeopathic Medical Society.* Held at Albany, N. Y., on Tuesday and Wednesday, Feb. 13 and 14, 1872.

*The Ethics of the Medical Profession.* Read before the Sacramento (Cal.) Society for Medical Improvement. By JOSEPH F. MONTGOMERY, M.D.

*Irrigations of Ice Water as a Means of Arresting Hæmorrhage in Cases of Placenta Prævia.* By W. O. BALDWIN, M.D., Montgomery, Ala. 1872.

*Lithotomy and Lithotrity.* Illustrated by Cases in the Practice of GURDON BUCK, M.D., Visiting Surgeon to the New York Hospital and Presbyterian Hospital, etc. New York, 1872.

